# Thoracic Actinomycosis: A Rare Cause of Pleural Thickening

**DOI:** 10.1002/rcr2.70451

**Published:** 2026-04-13

**Authors:** Evonne Shum, Jaidyn Muhandiramge, Jay Finn, Jasmine Zhu, Rhoda Cameron, Ronan O'Donnabhain

**Affiliations:** ^1^ Department of General Medicine Austin Hospital Melbourne Victoria Australia; ^2^ School of Public Health and Preventive Medicine Monash University Melbourne Victoria Australia; ^3^ Department of Pathology Austin Hospital Melbourne Victoria Australia

**Keywords:** actinomycosis, infection, malignancy, pleural

## Abstract

A middle‐aged man presented with an indolent growth on his chest wall, features of sepsis, and constitutional symptoms. He was subsequently found to have raised inflammatory markers and pleural thickening on imaging which, coupled with a history of asbestos exposure, was concerning for malignancy. While there was clinical improvement with empirical intravenous penicillin antibiotics, the diagnosis was not certain until an excisional biopsy of the mass was performed, with histopathology showing Splendore Hoeppli material (‘sulfur granules’) leading to the diagnosis of an Actinomyces infection. The diagnosis allowed for appropriate management with a prolonged course of antibiotics, resulting in complete resolution of symptoms. This case highlights the importance of investigation for atypical infection in patients with pleural thickening, particularly in those with risk factors such as drug use.

## Graphical abstract

We report a case of a man who presented with a chest wall mass and was found to have pleural thickening on PET, with features concerning for mesothelioma. Further investigation revealed a diagnosis of actinomyces, a rare infectious cause of pleural thickening.

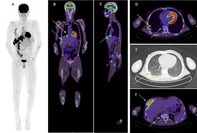

## 1 Introduction

Actinomycosis is a rare infection caused by filamentous Gram‐positive anaerobic bacteria from the Actinomycetaceae family [1]. Misdiagnosis is common as it can mimic malignancy or other granulomatous disease such as tuberculosis [1]. Risk factors associated with actinomycosis include age between 20 and 60 years, male sex, diabetes, and immunosuppression [1]. Thoracic actinomycosis may present as bronchopulmonary or pleural disease, with the latter sometimes mimicking mesothelioma, but can also extend to the chest wall [2]. Once diagnosed, it is curable with prolonged antibiotics, usually penicillin, though surgery may be required [1].

## 2 Case Report

A 47‐year‐old male was transferred from a regional hospital to a metropolitan hospital for investigation of a systemic illness manifested by a painful chest wall lump, a rash, and constitutional symptoms including fevers, malaise, anorexia, and 15 kg of weight loss. Initial investigations showed raised inflammatory markers and possible pleural lesions on CXR, necessitating transfer for a biopsy to investigate for malignancy. The chest wall mass had been increasing in size since its onset 4 weeks prior. The patient's past medical history was significant for untreated hepatitis C infection. He was an active user of intravenous drugs, having recently injected heroin 1 week prior to admission and marijuana via a bong. He reported significant previous asbestos exposure, reporting having played with it as a child. He lived at home with his mother, worked as a poultry technician and had had no recent travel.

On arrival, he was febrile, tachycardic, and hypotensive (BP 101/70 mmHg). Examination revealed a 4 × 10 cm tender, erythematous, fluctuant mass over the right anterolateral 6–9th ribs.

Blood tests revealed a normocytic anaemia with a haemoglobin of 89 g/L. Leukocytes, platelets, renal and liver function were normal. Inflammatory markers were elevated with a C‐reactive protein of 145 mg/L and erythrocyte sedimentation rate of 88 mm/h. The patient was hepatitis C positive with known, untreated infection, but had tested negative for HIV and tuberculosis (via the QuantiFERON‐TB GOLD assay).

**FIGURE 1:**
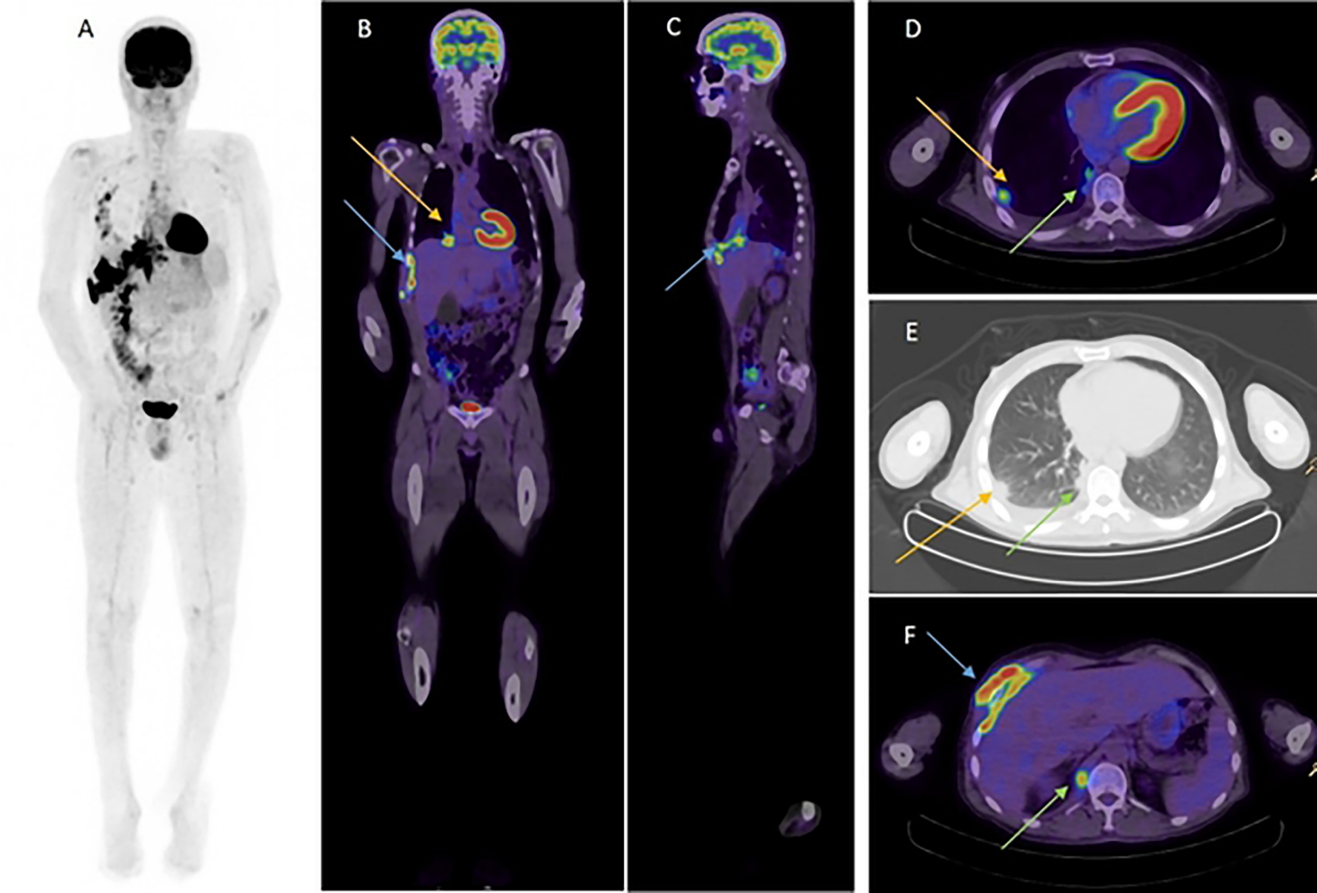
Image A shows whole body maximum intensity projection (MIP) used in PET imaging to visualise and represent a 3D distribution of radiotracer uptake in the body on a 2D plane. Image B, C, D and F are fused PET/CT images in the coronal, sagittal, transaxial and transaxial views respectively, whereas image E is a low dose CT transaxial view. Blue arrows demonstrate intense abnormal FDG uptake in the right hemithorax with involvement of the chest wall. Orange arrows demonstrate a 6 mm FDG avid right lower lobe lung nodule with associated right pleural effusion seen on the transaxial views. Green arrows demonstrate a FDG avid right distal para‐oesophageal node in the posterior mediastinum.

A CT chest revealed a mass‐like soft tissue surrounding the anterior and lateral aspects of the lower right ribs involving the superficial, intercostal and extrapleural space deep to the ribs extending into the right pericardiophrenic recess. There was also medial pleural thickening and reactive pericardial effusion. An FDG‐PET CT was obtained to identify a suitable (and metabolically active) lesion for biopsy and demonstrated intense abnormal FDG uptake in the right hemithorax, pleural and mediastinal nodes, without contralateral chest or distant involvement (Figure 1).

Given the patient's asbestos exposure and pleural changes, mesothelioma was a key differential. Other differentials included primary soft tissue tumours (e.g., sarcoma, extra‐abdominal desmoid tumour, and primary bone tumour), metastases, or direct extension of a lung tumour. The definitive diagnosis for any malignancy would require tissue diagnosis. Given the patients' infective symptoms and response to antibiotics, a cutaneous infection or infected cystic lesion was also considered. Q fever, an atypical zoonotic disease caused by *Coxiella burnetii* , was considered given the patients occupation and respiratory symptoms, although a chest wall mass would be atypical. Similarly, histoplasmosis, a fungal infection caused by histoplasma capsulatum, can be transmitted by bird droppings, although its cutaneous manifestations tend to be immune‐mediated and widespread in nature, although discrete nodules are possible. Tuberculosis, with pulmonary and cutaneous manifestations, was also considered, although the patient had no risk factors for infection. Organisms of the order Actinomycetales (including Actinomyces and Nocardia) also have the potential to cause both cutaneous and pulmonary infection. Ultimately, culture was needed for diagnosis, but no exudate was present; biopsy was thought more useful.

**FIGURE 2:**
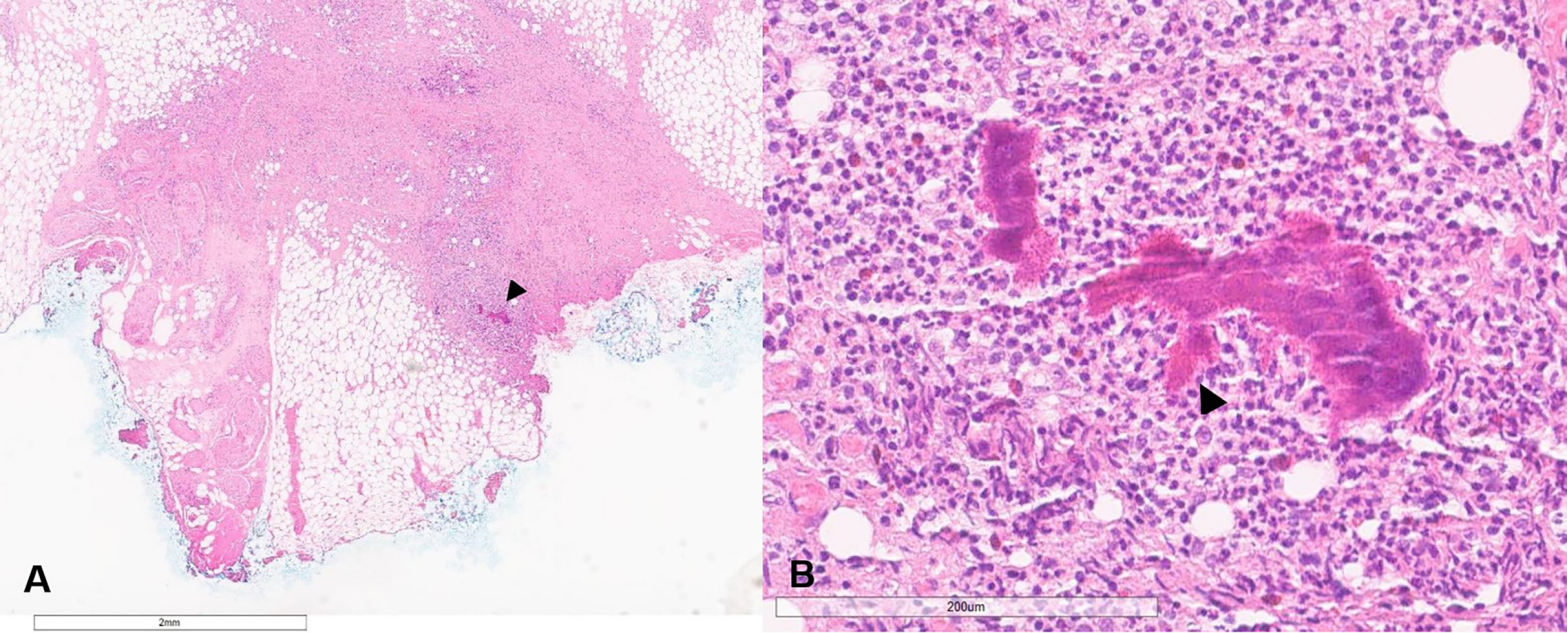
Image A shows a low‐power view of the right chest wall excisional biopsy. There is subcuticular tissue with fibrosis and mixed inflammation. The arrowhead points to a cluster of ‘sulfur granules’. Image B shows a high‐power view of the ‘'sulfur granules’, comprising large colonies of basophilic organisms with surrounding eosinophilic, Splendore‐Hoeppli material (arrowhead). There is suppurative inflammation in the background, with large numbers of neutrophils, as well as intermixed eosinophils and histocytes.

The patient underwent an ultrasound‐guided core biopsy of the right chest wall mass, but histopathology demonstrated no microorganisms on staining and no neoplastic process. He subsequently had an excisional biopsy of the chest wall mass, which showed mixed suppurative and chronic panniculitis with focal colonies of basophilic organisms with surrounding Splendore Hoeppli material (‘sulfur granules’) on histopathology (Figure 2). This histopathological finding can be caused by bacteria of the Actinomycetales order (e.g., Actinomyces, Nocardia), Botyromycosis, and fungal infection (eumycotic mycetoma).

The patient had received intravenous (IV) flucloxacillin, IV ceftriaxone and IV amoxicillin/clavulanic acid on the day of transfer, with the latter continued for eight days total while the above investigations were taking place. The patient demonstrated a marked symptomatic response to the IV amoxicillin/clavulanic acid, with rapid resolution of his constitutional symptoms.

Given the imaging findings, symptoms, and history of asbestos exposure, the patient was initially investigated for mesothelioma, although atypical infection was considered. The discovery of Splendore Hoeppli material on histopathology, in conjunction with rapid response to amoxicillin (given other potential causative organisms would not have responded to penicillin), led to a presumptive diagnosis of pulmonary actinomycosis (with extrapulmonary dissemination) being made. The likely source was contaminated water from the patient's water pipe given his infrequent cleaning of the paraphernalia.

Following diagnosis of actinomycosis, the patient was commenced on oral amoxicillin 1 g three times daily with a view to continue treatment for at least 6 months as per the Australian Therapeutic Guidelines. He was reviewed 2 weeks post‐discharge in the Infectious Diseases Outpatient Clinic and had responded well to amoxicillin treatment. The patient did not attend further clinic appointments or imaging but reported resolution of symptoms one year post‐treatment on phone follow‐up.

## 3 Discussion

This case study describes a case of thoracic actinomycosis in a high‐risk patient and highlights the importance of concurrent investigation for atypical infection such as actinomycosis in patients with pleural thickening, particularly if they have risk factors that increase the level of suspicion for an infection. In this case, while the patient's presentation was highly suspicious for mesothelioma given his previous asbestos exposure and pleural thickening on imaging, further investigations, particularly a tissue biopsy, were required to reveal the diagnosis of actinomycosis. The patient's frequent use of marijuana via a water pipe and a rapid clinical response to antibiotics were two factors that may have raised the suspicion of an atypical infection.

Actinomycosis is an anaerobic gram‐positive bacilli bacterial infection, generally localised in the cervicofacial region, but also found in the abdominal and thoracic regions [3]. The bacteria itself are a commensal of the oropharynx, gastrointestinal system and female genitalia, acting opportunistically through breakdowns in mucosa [4]. Risk factors for pathogenic infection include poor dentition and intravenous drug use, with alcoholism, diabetes mellitus and chronic lung disease being specific risk factors for thoracic infection [3, 5]. Overall prevalence of the disease is 1 in 300,000, with roughly a 3:1 ratio of men to women being affected [5, 6]. Early stages of pulmonary/thoracic actinomycosis are generally characterised by a slow‐growing peripheral fibrotic lung mass, potentially with cavitation or invasion of local tissue, often mistaken for malignancy in early stages [5, 7]. Extrapulmonary spread to the mediastinum, pleura and chest wall is also possible, as was seen in this clinical case in the form of a chest wall mass, with less common routes of spread being haematogenous or spread from known cervicofacial infection to the thorax [7]. Common presenting complaints include mild fever, weight loss, productive cough, haemoptysis, dyspnoea or chest pain, with common differential diagnoses being chronic lung infections such as tuberculosis or lung malignancy [8]. Progression of the fibrotic lung lesion and subsequent cavitation can lead to worsening respiratory function and purulent discharge [9].

The presence of Splendore Hoeppli material (which resemble ‘sulfur granules’) and filamentous gram‐positive fungal appearing pathogens on gram or histological staining is suggestive of Actinomyces infection although they can also be seen in other bacteria of the Actinomycetales order (e.g., nocardia), botryomycosis, and fungi (eumycotic mycetoma) [3, 10]. Specific culturing of the organism through anaerobic culture is complicated by the presence of contaminant organisms, prior antibiotic therapy, or inadequate culture time, with growth on culture taking between 5 and 20 days [10, 11]. Of particular importance is sampling appropriate sites when clinical presentation is suggestive of Actinomycosis, ideally from biopsy and not from swabs, given the presence of the organism as a commensal in mucosa.

The diagnosis can also be obtained via imaging findings suggestive of the fibrosing granulation tissue characteristic of the infection [2]. With the potential for actinomycosis to mimic malignancy or other granulomatous disease such as tuberculosis [3], it is not uncommon for a PET‐CT scan to be performed such as in this clinical case, and while not necessary for diagnosis, it can be helpful in identifying an appropriate site for biopsy. In such circumstances, the FDG avid region may appear similar to that expected in malignancy [12]. It should be noted that in our center, PET scans are very accessible and are not uncommonly used in the investigation of atypical infection and malignancy. Ultimately, the gold standard for diagnosis is via a tissue biopsy for culture and histopathology [12, 13]. With regards to thoracic actinomycosis infection and its potential for pleural and chest wall invasion, early CT scans can be helpful for identifying target regions for biopsy, and potentially showing signs of consolidation, cavitation, ground glass opacity, and pleural effusion [5, 8]. Currently, the use of bronchoscopy is primarily targeted at ruling out malignancy and has less utility in the diagnosis of actinomycosis [7, 9].

Current treatment guideline recommendations are for prolonged antibiotic therapy, generally with 6–12 months of penicillin, usually amoxicillin [14]. The duration of treatment is often individualised and depends on initial burden of disease, degree and success of surgical resection (if required), and intermediate response to treatment [15]. Surgery may be required to exclude malignancy or if large abscesses cannot be drained by percutaneous aspiration [15]. The various strains of Actinomyces are generally susceptible to beta‐lactams, making amoxicillin the treatment of choice. The pathogen does not have capacity for production of beta‐lactamases, and as such, the use of clavulanic acid in addition to amoxicillin in monomicrobial Actinomyces infection is not recommended [10]. Notably, in this case, response to penicillin antibiotics assisted with diagnosis and differentiating actinomycosis from other ‘sulfur granule’‐associated infections.

## Author Contributions

**Evonne Shum:** conceptualization, data curation, methodology, investigation, project administration, visualisation, writing – original draft, and writing – review and editing. **Jaidyn Muhandiramge:** conceptualization, data curation, methodology, investigation, project administration, visualisation, writing – original draft, and writing – review and editing. **Jay Finn:** conceptualization, data curation, methodology, investigation, project administration, visualisation, writing – original draft, and writing – review and editing. **Jasmine Zhu:** data curation, investigation, visualisation, and writing – review and editing. **Rhoda Cameron:** data curation, investigation, visualisation, and writing – review and editing. **Ronan O'Donnabhain:** conceptualization, data curation, methodology, investigation, project administration, visualisation, writing – original draft, and writing – review and editing.

## Consent

The authors declare that written informed consent was obtained for the publication of this manuscript and accompanying images and attest that the form used to obtain consent from the patient complies with the Journal requirements as outlined in the author guidelines.

## Conflicts of Interest

The authors declare no conflicts of interest.

## Data Availability

The data that support the findings of this study are available on request from the corresponding author. The data are not publicly available due to privacy or ethical restrictions.
